# Development and evaluation of an ultra-high-resolution cone-beam computed tomography system for hand and foot

**DOI:** 10.1007/s00256-025-05077-z

**Published:** 2025-11-13

**Authors:** Wakiko Tani, Katsuhiro Ichikawa, Hiroki Kawashima, Kaoru Tada

**Affiliations:** 1 Center for Radiology and Radiation Oncology, Kobe University Hospital, 7-5-2 Kusunoki-cho, Chuo-ku, Kobe, Hyogo 650-0017 Japan; 2 Division of Health Sciences, Graduate School of Medical Science, Kanazawa University, 5-11-80 Kodatsuno, Kanazawa, 920-0942 Japan; 3 Faculty of Health Sciences, Institute of Medical, Pharmaceutical and Health Sciences, Kanazawa University, 5‑11‑80 Kodatsuno, Kanazawa, Ishikawa 920‑0942 Japan; 4 Department of Orthopaedic Surgery, Kanazawa University, 13-1 Takaramachi, Kanazawa, 920-8641 Japan

**Keywords:** Cone-beam computed tomography, Extremity, High resolution, Spatial resolution, Radiation dose, Image noise

## Abstract

**Objective:**

To develop an experimental ultra-high-resolution cone-beam computed tomography (UHRCBCT) system for imaging the human hand and foot and to evaluate its spatial resolution, image noise, radiation dose, and feasibility through phantom and volunteer studies.

**Materials and methods:**

The UHRCBCT system was built based on a 0.5-mm focal spot X-ray tube and a 0.099-mm pixel CMOS detector. A low magnification geometry (magnification: 1.22) was applied to reduce geometry blurring. Images were reconstructed with a voxel size of 0.10 × 0.10 × 0.10 mm^3^. Radiation dose was quantified by measuring the CT dose index (CTDI) corresponding to the scanning method. Spatial resolution and image noise were quantitatively evaluated using a 0.1-mm copper wire and a water phantom, respectively, in comparison with those of a high-resolution multi-slice CT system (HRMSCT, detector size: 0.25 mm at isocenter) for the matched radiation dose. Scans were also performed on a foot phantom using UHRCBCT and HRMSCT and on carpal bones of healthy volunteers using UHRCBCT under ethical approval.

**Results:**

The effective dose estimated from CTDI was 0.024 mSv using a conversion factor of 0.0005 mSv/(mGy cm), which was within the lowest category for healthy volunteers in ICRP publication 103. The 5%MTF-based spatial resolution was 0.10 mm, significantly smaller than 0.23 mm achieved by HRMSCT. Image noise was approximately twice as high in UHRCBCT; however, trabecular bone structures and joint spaces were more distinctly visualized in both phantom and volunteer images.

**Conclusion:**

UHRCBCT achieved a high resolution of 0.10-mm in imaging trabecular hand and foot bones with an acceptable radiation dose, indicating potential for clinical application in orthopedic diagnosis.

## Introduction

In recent years, cone-beam X-ray computed tomography (CBCT) has emerged as a promising modality for foot and hand fractures and weight-bearing imaging of foot to knee [1–5]. While it holds promise for application in clinical situations which demand higher resolution with lower radiation dose and higher speed, there still seems to be some room for improvement before gaining wider acceptance in terms of image resolution and practicality in clinical use.

First, CBCT is still short of delivering high enough resolution for certain clinical situations. It is often reported to be superior to conventional radiography and MRI in accurately visualizing fine bone structural changes, especially in regions with complex anatomy [4, 6, 7]. However, to our best knowledge, only a limited number of CBCT systems have been commercially available for application to extremities. Despite their vendors’ claims of high-resolution imaging, their actual spatial resolution is rather limited to approximately 0.3 to 0.5 mm [1, 3, 8, 9]. While higher than that of conventional clinical multi-slice CT systems, it is still not high enough to effectively visualize bone trabeculae, which typically measure about 0.1 to 0.2 mm [10–12]. The capability to visualize fine trabecular structures may further enhance the assessment of subtle fractures, early degenerative changes, and bone quality. Such detailed visualization could complement existing imaging modalities and expand the potential clinical applications of extremity CBCT. CBCT systems generally also have lower contrast resolution than conventional CT; thus, strategies for improving the contrast resolution such as the implementation of an anti-scatter grid should be considered.

While weight-bearing CT based on CBCT is generally effective for the assessment of joint spaces, alignment, and load-dependent changes in bone morphology, emphasis has been placed on the scanner’s ability to perform the posture with good enough resolution and low radiation dose, rather than maximizing image sharpness to visualize bone microstructures. Whereas a recent paper reports the application of an experimental CBCT system based on a high-resolution X-ray detector (pixel size: 0.099 mm) to human extremities (not a bench-top system with a rotating table), its application is limited to the tibia of human cadavers; furthermore, the spatial resolution is not explicitly stated [13].

Another issue is the relatively long scan time even for obtaining motionless images. A recent high-resolution peripheral quantitative CT (HR-pQCT), which is reported to provide significantly high-resolution images, requires a scan time of 2 min with a z-directional coverage of only approximately 10 mm. Obviously, this would be too long for most patients. For CBCT to be of any clinical use, it needs to reduce the scan time down to a more acceptable level with a reasonable level of radiation dose.

The purpose of this study is therefore to develop an experimental ultra-high-resolution CBCT (UHRCBCT) system specifically applicable to the human hand and foot, with an emphasis on clinical practicality. The device we have developed offers a high spatial resolution of 0.08 mm (voxel size) at the rotation center and a short scan time of less than 10 s. We first evaluated physical image quality and performance using a bone-embedded phantom and subsequently assessed near-clinical performance by imaging the carpal bones of healthy volunteers under the approval of our institutional ethical review board.

## Materials and methods

### UHRCBCT system

**Fig. 1:**
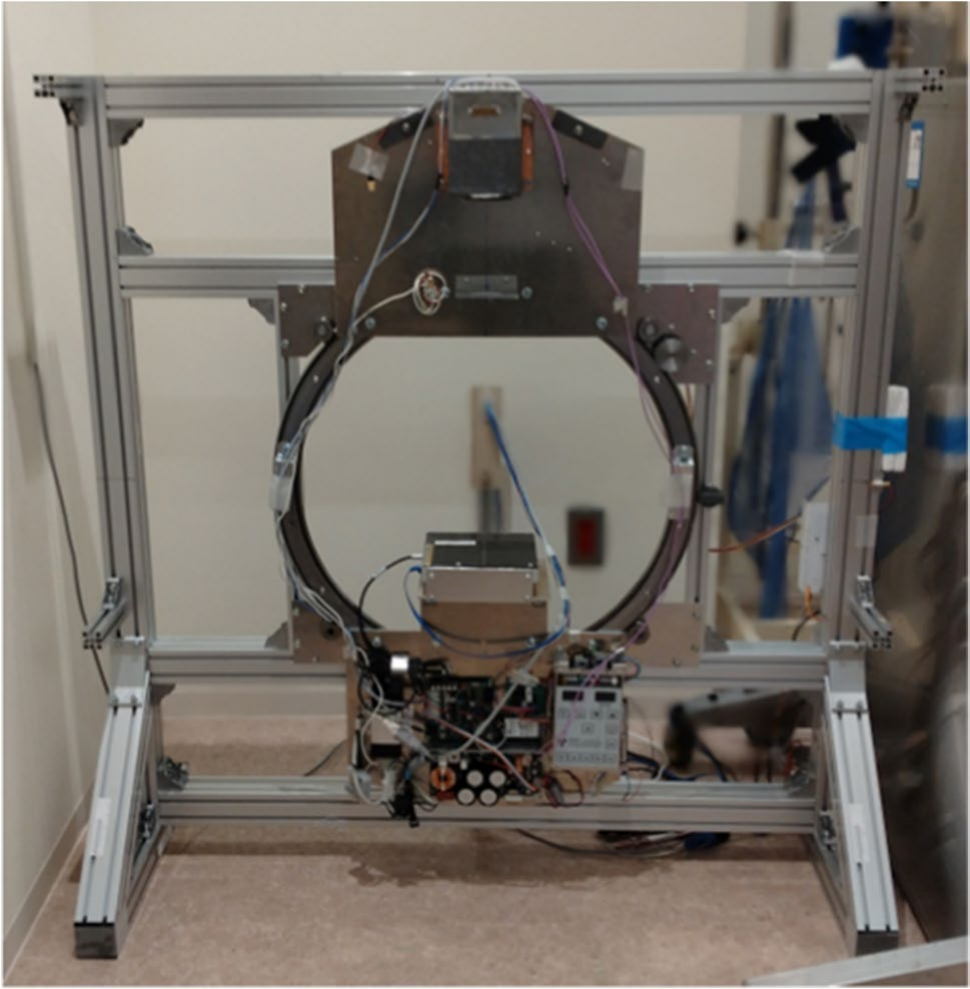
An ultra-high-resolution cone-beam CT system (UHRCBCT) for hand and foot we developed

We constructed the UHRCBCT system consisting of five main parts: an X-ray unit, a detector unit, a ring rail, a motor drive unit, and a computer (Fig. 1). The detector is a CMOS detector with a pixel size of 0.099 mm (Xineos 1511, Teledyne DALSA, Eindhoven, NL). The X-ray unit is for a mobile unit with a 0.5-mm focal spot, a maximal tube voltage of 100 kV, and a maximal tube current of 10 mA (DXR-100, Mikasa X-ray corporation, Tokyo, Japan) with continuous X-ray. The X-ray unit and the detector are mounted on a ring rail with an inner diameter of 612 mm (R44-612P, HepcoMotion, Tiverton, Devon, UK) using hand-made supports made of aluminum. This assembly is rotated, using a high-power stepper motor. A custom-made fiber interspace anti-scatter grid provided by Mitaya corporation, Kawagoe, Saitama, Japan, is placed on the detector. This one-dimensional grid is focused according to the geometry of the system. The preliminarily measured Buck factor was 2.66. Using this system, we were able to calculate CT numbers more precisely than using commercial CBCT systems, which are generally equipped with no anti-scatter grids. The scan condition for the adult hand was set to 80 kV, 7 mA, 6.5 s per 360-degree rotation.

### Geometry for high resolution

In the UHRCBCT we developed, to achieve ~0.1 mm resolution, a low magnification geometry known to significantly reduce X-ray focus penumbra (i.e. the geometry blurring) was adopted to fully utilize the high resolution potential of 0.099 mm pixel of the CMOS detector. The distances from the focal point to the rotation center and from the rotation center to the detector surface were 470 mm and 105 mm, respectively; with these, the magnification was 1.22, and the estimated focus penumbra size was 0.09 mm.

### Image reconstruction

CT images were reconstructed using the Feldkamp method [14], which is known to be the standard technique for CBCT reconstruction and no noise reduction algorithm was applied. The x–y and z-directional coverages were 122.0 mm and 51.2 mm at maximum, respectively, Within the coverage, fundamentally, volume data with 512 × 512 × 512 voxels with a voxel size of 0.10 × 0.10 × 0.10 mm^3^ was generated, and the matrix size can be expanded up to 1220 × 1220 × 512 voxels depending on target sizes. We limited the z-directional coverage to shorten the scan time (by shortening the data acquisition time for each view) for reducing the possibility of occurring blurring by the motion. The z-directional coverage can be expanded up to 90 mm using the detector’s full coverage. A high-resolution reconstruction filter kernel with no edge-enhancement characteristic was used to obtain high resolution and to prevent unnecessary CT number alternations (i.e., undershoot and overshoot).

### Physical performance measurement

#### Comparator

It was difficult to use a commercial CBCT system for comparison because of its very limited availability in Japan; instead, we used a high-resolution whole-body multi-slice CT (HRMSCT) system with a detector pitch of 0.25 mm (Aquilion Precision, Canon medical, Japan) which has been reported to provide a high resolution of 0.2 mm (5%MTF of 2.55 mm^−1^) [15]. The resolution is higher than that of commercial CBCT systems for extremities (0.3 to 0.5 mm as mentioned earlier). The FC86 reconstruction kernel was selected because it has the highest frequency limit among kernels with high resolution and no edge enhancement. The slice thickness was set to the minimum one of 0.25 mm. A total of 512 slices, each with 512 × 512 pixels and a pixel size of 0.10 × 0.10 mm^2^, were reconstructed with an increment of 0.10 mm. Thus, a total of 512 × 512 × 512 voxels were generated with the same nominal voxel dimensions as in UHRCBCT’ s reconstruction.

#### Dose measurement

The CT dose index (CTDI) measurements for UHRCBCT were performed using a dose analysis system (Radcal ACCU-GOLD+, Radcal Corporation, Monrovia, CA, USA) in combination with a 0.6 cc ionization chamber (10X6-0.6CT, Radcal Corporation). The chamber was positioned at the center and peripheral locations of a 16 cm diameter standard CTDI phantom. The CTDI of HRMSCT was obtained from the value displayed on its operation console (i.e., the dose was not measured). The console display system had been calibrated within 1 year prior to this study through the institutional quality assurance procedure for CT dosimetry.

### Spatial resolution

To quantitatively evaluate the spatial resolution, the wire method [15–19] was used with a 0.10-mm copper wire enclosed in a cylindrical water phantom with a diameter of 50 mm. The modulation transfer function (MTF) was calculated from the cross-section CT image. For UHRCBCT, the scanning condition was the same as the baseline (80 kV, 7.0 mA, 6.5 s). For the HRMSCT, the scanning parameters were adjusted to 80 kV, 200 mA, 1.50 s/rotation, and a helical pitch factor of 0.56 so that the CTDI value displayed on the operator console matched the measured dose of the UHRCBCT. A software program called CTmeasure (version 0.99d), which is provided by the Japanese Society of CT Technology and was previously validated for accuracy in a study [19], was used to calculate MTF.

### Image noise

A cylindrical water phantom with a diameter of 9.0 cm was scanned under the same conditions as for spatial resolution. The noise power spectrum (NPS) of the CT images was measured using a two-dimensional Fourier transform method [15–17, 19]. CTmeasure was also used in this evaluation. The purpose of the NPS measurement was to confirm the effect of the difference in z-directional detector width (0.08 mm for UHRCBCT vs. 0.25 mm for HRMSCT), rather than to investigate whether the noise level of UHRCBCT is comparable to that of HRMSCT. It is naturally conceivable that as the detector width in the z-direction decreases, the image noise increases because of the lower dose per slice.

### Phantom scanning

**Fig. 2:**
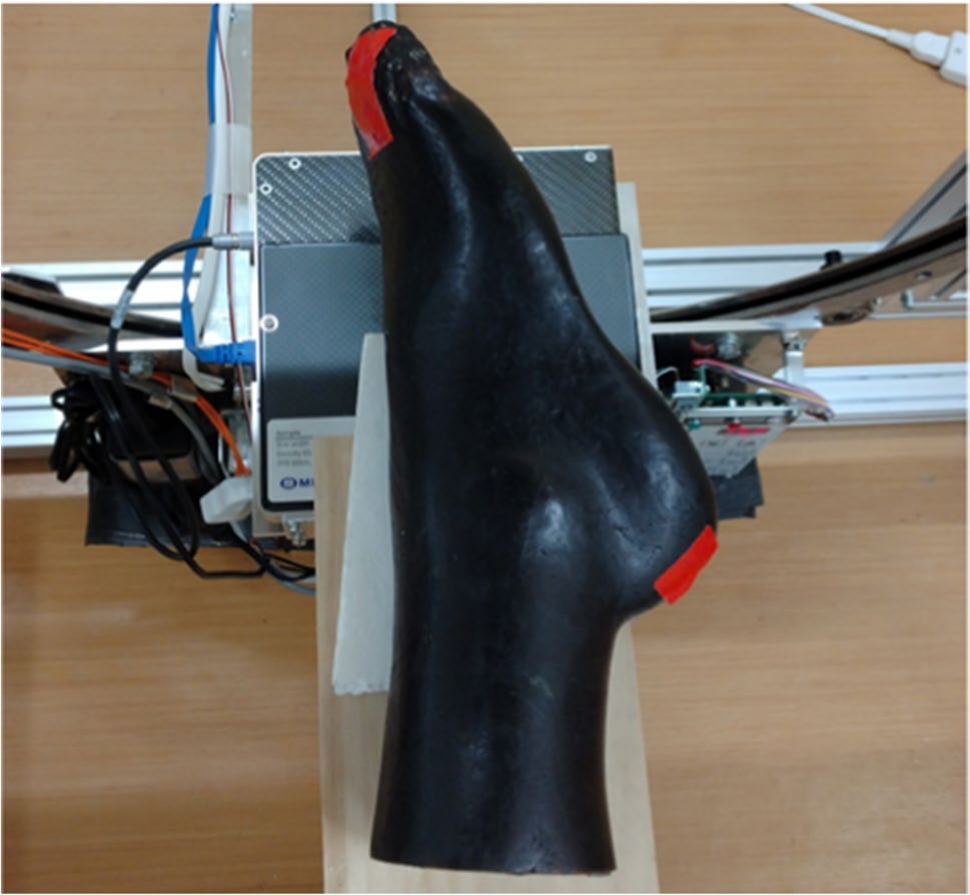
The foot phantom and its placement during the scan

An anthropomorphic foot phantom including dry human bones (source unknown) (Fig. 2) was scanned using the two systems under the same conditions as in the two preceding measurements.

### Scanning healthy volunteers

The carpal bones of two healthy volunteers, aged 62 and 58, were scanned using UHRCBCT. The study protocol was reviewed and approved by the institutional ethical review board (approval number: 1005). Written informed consent was obtained from each participant after providing a detailed explanation of the study purpose and the estimated radiation dose, which corresponds to the lowest dose criterion (< 0.1 mSv) defined for healthy volunteers in a relevant international guideline, ICRP Publication 103. To minimize hand motion during the scan, each volunteer’s hand was gently secured to the scanning table using an elastic band.

## Results

### Radiation dose

The measured CTDI for UHRCBCT was 9.28 mGy, and the corresponding effective dose was estimated to be 0.024 mSv from the dose length product of 47.5 mGy-cm (9.28 × 5.12) and the organ-specific conversion factor of 0.0005 mSv/(mGy cm) for the extremities [20]. As described above, the scanning condition for the HRMSCT was determined so that the CTDI value displayed on the operator’s console would closely match this CTDI value measured for the UHRCBCT.

### Spatial resolution

**Fig. 3:**
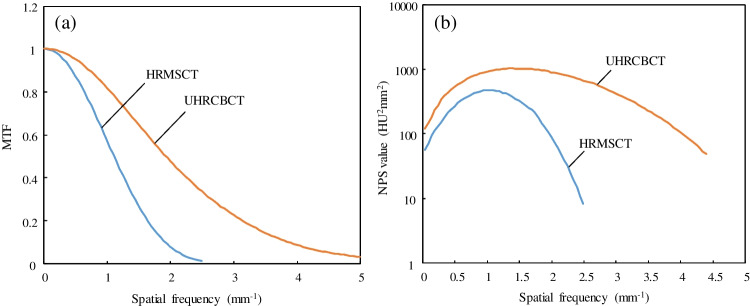
Measurement results: **a** modulation transfer function (MTF) and **b** noise power spectrum (NPS) for UHRCBCT and HRMSCT (with a 0.25-mm detector)

Figure 3a shows the measured MTFs for UHRCBCT and HRMSCT. The 10%MTF and 5% MTF were 4.14 and 4.85 mm^−1^ for UHRCBCT 1.91 and 2.15 mm^−1^ for HRMSCT. As a result, the resolutions estimated from the 5%MTFs were calculated to be 0.10 mm and 0.23 mm, respectively, using the formula introduced in [21].

### Noise power spectrum

Figure 3b shows the results of the NPS measurements. The noise level was remarkably higher with UHRCBCT than with HRMSCT. The increase factor of UHRCBCT over HRMSCT was 2.0 to 2.3 at low spatial frequencies of 0.1 to 0.3 mm^−1^. It should be noted that noise should be comparatively evaluated at spatial frequencies as low as this level, where MTF is similar between UHRCBCT and HRMSCT and hence the noise levels are comparable.

### Phantom image

**Fig. 4:**
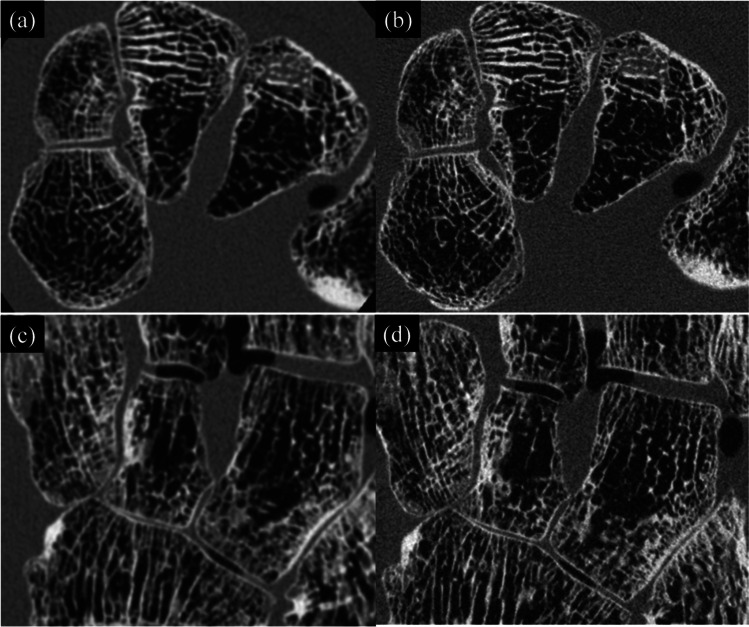
Axial and coronal multi-planar reformation (MPR) images: **a** axial, HRMSCT; **b** axial, UHRCBCT; **c** coronal, HRMSCT; and **d** coronal, UHRCBCT, corresponding to the foot phantom presented in Fig. 2. Within the reconstructed matrix of 512 × 512, regions with 450 × 350 pixels are presented here to emphasize the differences in spatial resolution. The window width/center was set at 3000/700 HU

Figure 4 shows axial and coronal multi-planar reformation (MPR) images. Not only was the trabecular bone structure rendered much more sharply, but also the subtle joint space with UHRCBCT than with HRMSCT. While the observed noise level was higher for UHRCBCT than for HRMSCT, this shortcoming was more than compensated for by the sharpness of the resulting image.

### Image of healthy volunteer

**Fig. 5:**
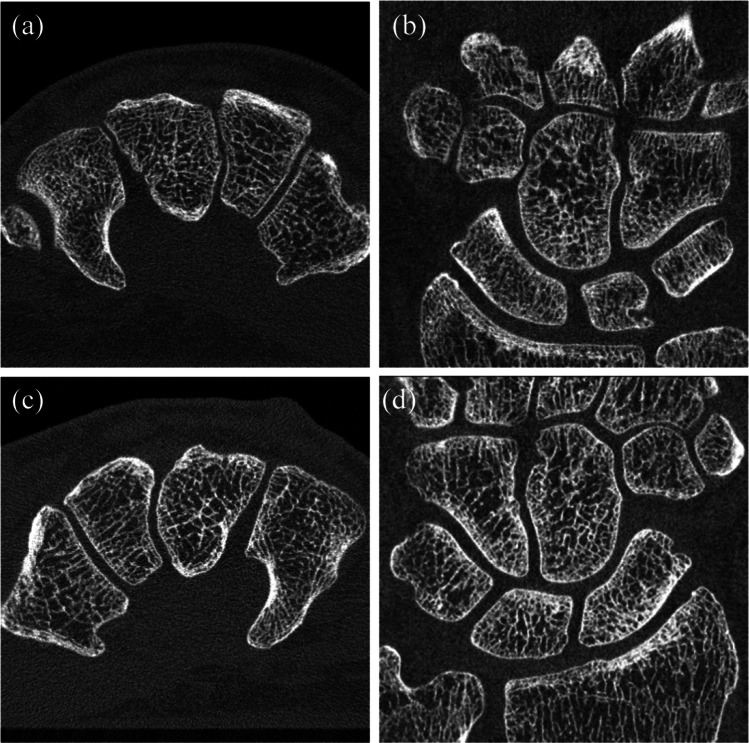
Multi-planar reformation (MPR) images of human extremity bones: (**a)** axial and (**b**) coronal, volunteer 1; (**c)** axial, volunteer 2; and (**d)** coronal, volunteer 2, with UHRCBCT. Scans were performed after the approval of the institutional ethics review board. The window width/center was set at 3000/700 HU

**Fig. 6:**
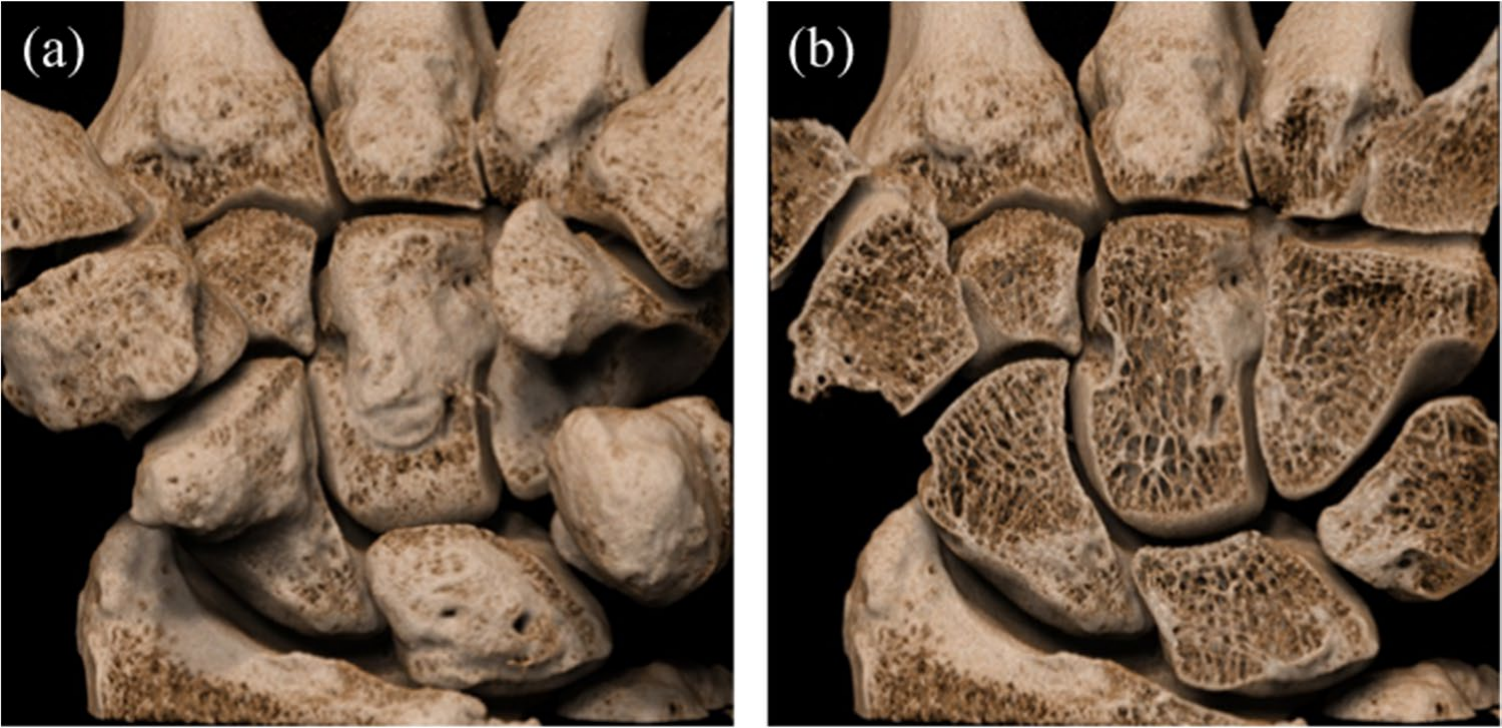
Photorealistic three-dimensional (3D) images for the healthy volunteer corresponding to Fig. 5(**a** and **b**), (**a)** without and (**b)** with coronal cutting, generated using a photo-realistic volume rendering application

Figure 5 shows axial and coronal MPR images of the carpal bones of healthy volunteers. They all render fine bone structures and joint spaces as clearly as the phantom images. No motion artifacts can be found in any of them. Figure 6 shows three-dimensional (3D) images generated using a photorealistic volume rendering application (Cinematic VRT, Siemens Healthineers). They, too, render the fine bone structure (including its trabeculae), and its three-dimensional relationship with the surroundings with such precision and fidelity that they appear almost as if one were observing an anatomical specimen.

## Discussion

We have developed an ultra-high-resolution CBCT system (UHRCBCT) capable of scanning human hands and feet with a voxel size of 0.08 mm. The measured spatial resolution was 0.10 mm, which was obviously smaller than 0.23 mm measured with MSHRCT and 0.3 to 0.5 mm reported with some existing commercial CBCT systems for extremities. The noise level increased by factors of 2.0 to 2.3 because of UHRCBCT’ s smaller z-directional detector width. With UHRCBCT, the phantom images were significantly sharper than with HRMSCT, being consistent with the results of spatial resolution. CT scans of healthy volunteers produced CT images in which the bone trabeculae were clearly visualized with no motion artifacts. The estimated radiation dose is 0.024 mSv, which falls in the lowest category for healthy volunteers in ICRP Publication 103.

The effective dose of 0.024 mSv was within a reported CBCT dose range from 0.02 to 0.08 mSv for cases including wrist, ankle, and elbow [3]; on the other hand, it is higher than 0.01 mSv for ankle [22] and 0.013 mSv for knee [23]. These results suggest that CT images with an extremely high spatial resolution of 0.10 mm are obtainable even with doses not significantly higher than those reported here. The images in this study were reconstructed without using any noise reduction techniques; thus, dose reduction would not be impossible with the help of image-based techniques that have been proposed with clearly explained algorithms [24–26].

Our attempt to enhance spatial resolution through the use of low magnification geometry (magnification: 1.22) proved highly effective, achieving an exceptionally high resolution of 0.10 mm (5%MTF of 4.85 mm⁻^1^) and 0.12 mm estimated from the 10%MTF. Whereas the X-ray focus size we employed was 0.5 mm, which was not sufficiently small for high resolution imaging, the low magnification reduced its focus penumbra to as small as 0.09 mm and its blurring effect was nearly eliminated. Since the focal spot was not extremely small, we did not need to significantly limit the tube current. As a result, we were able to keep the scan time shortest, taking advantage of the full speed of the detector used. Although the low magnification resulted in a short distance between the rotation center and the detector surface, the gantry bore size was still approximately 200 mm, which was adequate to accommodate a human hand or foot. The current in-plane coverage is sufficient for imaging the elbow but sometimes insufficient for the knee. However, the 200-mm gantry bore is large enough to accommodate the knee, requiring only a larger detector to achieve full coverage. In this case, degradation of spatial resolution is expected to be limited to the peripheral regions, since an increase in magnification is not necessary. When imaging the knee, the tube current may need to be increased; however, the focal spot size of 0.5 mm is not particularly small, allowing the tube current to be raised up to 10 mA (approximately 1.4 times the current used for hand or foot imaging) and the tube voltage up to 100 kV with the present X-ray system.

With UHRCBCT, image noise was approximately twice as high as with HRMSCT. Although more noise was recognized in UHRCBCT’s images, UHRCBCT’s high spatial resolution enabled the detection of the fine structures that were lost in the lower-resolution images with HRMSCT. This advantage in anatomical detail effectively outweighs the drawback of increased noise.

The results of healthy volunteers’ images seem to indicate a growing clinical utility of UHRCBCT. The extremely high-resolution images have potential that facilitates evaluation not only of trabecular bone density and microarchitecture but also of cortical bone thinning and increased porosity. Therefore, it holds promise for application in degenerative disorders such as osteoporosis and osteoarthritis. Furthermore, it is expected to contribute to improved diagnostic accuracy in traumatic conditions such as microfractures. Although this particular system described here has currently no ability for weight bearing, UHRCBCT as a method can become a new diagnostic tool for hand and foot orthopedic thanks to the high resolution that has never been achieved before.

This study has several limitations. First, the effectiveness of a high resolution (0.10 mm) on diagnosis was not yet evaluated. Evaluations with clinical cases should be performed by radiologists and orthopedists in the future. Second, scan images with no motion artifacts were obtained with only two healthy volunteers. As UHRCBCT is very sensitive because of minuscule pixels, it should be confirmed that motion-artifact-less images could be steadily obtained for various cases including elderly patients and patients with pain at hand or foot. Third, the z-directional coverage was limited to 51.2 mm in this study, taking into account clinically acceptable scan lengths. Although the detector used is capable of extending the coverage up to approximately 90 mm, this would increase the scan time to approximately 10 s, making the acquisition more susceptible to motion artifacts. Finally, although the developed UHRCBCT system employs an anti-scatter grid to improve image contrast, cone-beam CT in general has inherently limited soft-tissue contrast. This limitation remains to some extent in our system because the available tube current is restricted by the X-ray unit used.

In conclusion, we have developed an experimental UHRCBCT system applicable to the human hand and foot. The measured spatial resolution was 0.10 mm, which has not been achieved before. Trabecular bone details in the carpal bones of healthy volunteers were clearly visualized with a low radiation dose of 0.024 mSv, which falls within the lowest dose category defined for healthy volunteers in existing guidelines.

## Data Availability

The dataset is available from the corresponding author upon reasonable request.

## References

[1] Koskinen SK, Haapamäki VV, Salo J, Lindfors NC, Kortesniemi M, Seppälä L, et al. CT arthrography of the wrist using a novel, mobile, dedicated extremity cone-beam CT (CBCT). Skeletal Radiol. 2013;42(5):649–57. 22990597 10.1007/s00256-012-1516-0

[2] Halonen KS, Mononen ME, Jurvelin JS, Töyräs J, Salo J, Korhonen RK. Deformation of articular cartilage during static loading of a knee joint–experimental and finite element analysis. J Biomech. 2014;47(10):2467–74. 24813824 10.1016/j.jbiomech.2014.04.013

[3] Huang AJ, Chang CY, Thomas BJ, MacMahon PJ, Palmer WE. Using cone-beam CT as a low-dose 3D imaging technique for the extremities: initial experience in 50 subjects. Skeletal Radiol. 2015;44(6):797–809. 25652734 10.1007/s00256-015-2105-9

[4] Gibney B, Smith M, Moughty A, Kavanagh EC, Hynes D, MacMahon PJ. Incorporating cone-beam CT into the diagnostic algorithm for suspected radiocarpal fractures: a new standard of care? AJR Am J Roentgenol. 2019;213(5):1117–23. 31287723 10.2214/AJR.19.21478

[5] Lôbo CFT, Bordalo-Rodrigues M, Group W-BCTIS. Weight-bearing cone beam CT scans and its uses in ankle, foot, and knee: an update article. Acta Ortop Bras. 2021;29(2):105–10. 34248411 10.1590/1413-785220212902236939 PMC8244836

[6] Suojärvi N, Haapamäki V, Lindfors N, Koskinen SK. Radiocarpal injuries: cone beam computed tomography arthrography, magnetic resonance arthrography, and arthroscopic correlation among 21 patients. Scand J Surg. 2017;106(2):173–9. 27456020 10.1177/1457496916659226

[7] Delsmann MM, Delsmann J, Jandl NM, Maas KJ, Beil FT, Amling M, et al. Advantages of cone beam computed tomography for evaluation of subchondral insufficiency fractures of the knee compared to MRI. Sci Rep. 2024;14(1):15278. 38961162 10.1038/s41598-024-64591-7 PMC11222521

[8] Neubauer J, Voigt JM, Lang H, Scheuer C, Goerke SM, Langer M, et al. Comparing the image quality of a mobile flat-panel computed tomography and a multidetector computed tomography: a phantom study. Invest Radiol. 2014;49(7):491–7. 24637586 10.1097/RLI.0000000000000042

[9] John Carrino, Eric Bogner, Cone beam CT: A technical explanation of imge quality characteristics, White paper of CurveBeam AI, 2024, Accessed on July 18, 2025

[10] Chiba K, Ito M, Osaki M, Uetani M, Shindo H. In vivo structural analysis of subchondral trabecular bone in osteoarthritis of the hip using multi-detector row CT. Osteoarthritis Cartilage. 2011;19(2):180–5. 21087677 10.1016/j.joca.2010.11.002

[11] Manske SL, Zhu Y, Sandino C, Boyd SK. Human trabecular bone microarchitecture can be assessed independently of density with second generation HR-pQCT. Bone. 2015;79:213–21. 26079995 10.1016/j.bone.2015.06.006

[12] Viero A, Biehler-Gomez L, Messina C, Cappella A, Giannoukos K, Viel G, et al. Utility of micro-CT for dating post-cranial fractures of known post-traumatic ages through 3D measurements of the trabecular inner morphology. Sci Rep. 2022;12(1):10543. 35732857 10.1038/s41598-022-14530-1 PMC9218115

[13] Subramanian S, Brehler M, Cao Q, Quevedo Gonzalez FJ, Breighner RE, Carrino JA, et al. Quantitative evaluation of bone microstructure using high-resolution extremity cone-beam CT with a CMOS detector. Proc SPIE Int Soc Opt Eng. 2019;10953. 10.1117/12.2515504 PMC6896315 31814656

[14] Feldkamp LA, Davis LC, Kress JW. Practical cone-beam algorithm. J Opt Soc Am A Opt Image Sci Vis. 1984;1(6):612–9.

[15] Kawashima H, Ichikawa K, Takata T, Nagata H, Hoshika M, Akagi N. Technical note: performance comparison of ultra-high-resolution scan modes of two clinical computed tomography systems. Med Phys. 2020;47(2):488–97. 31808550 10.1002/mp.13949

[16] Fujimura I, Ichikawa K, Miura Y, Hoshino T, Terakawa S. Comparison of physical image qualities and artifact indices for head computed tomography in the axial and helical scan modes. Phys Eng Sci Med. 2020;43(2):557–66. 32524440 10.1007/s13246-020-00856-5

[17] Hara T, Ichikawa K, Sanada S, Ida Y. Image quality dependence on in-plane positions and directions for MDCT images. Eur J Radiol. 2010;75(1):114–21. 19410407 10.1016/j.ejrad.2009.03.060

[18] Matsuura K, Ichikawa K, Kawashima H. Task-specific spatial resolution properties of iterative and deep learning-based reconstructions in computed tomography: comparison using tasks assuming small and large enhanced vessels. Phys Med. 2022;95:64–72. 35123172 10.1016/j.ejmp.2022.01.009

[19] Inoue T, Ichikawa K, Hara T, Ohashi K, Sato K, Kawashima H. Validating computer applications for calculating spatial resolution and noise property in CT using simulated images with known properties. Radiol Phys Technol. 2024;17(1):238–47. 38198065 10.1007/s12194-023-00771-w

[20] Joemai RM, Zweers D, Obermann WR, Geleijns J. Assessment of patient and occupational dose in established and new applications of MDCT fluoroscopy. AJR Am J Roentgenol. 2009;192(4):881–6. 19304690 10.2214/AJR.08.1765

[21] Hsieh J. High-Contrast Spatial Resolution. In: Hsieh J, editor. Computed tomography: principles, design, artifacts, and recent advances. 2nd ed. Bellingham (WA): SPIE Press; 2009. p. 143–50.

[22] Collan L, Kankare JA, Mattila K. The biomechanics of the first metatarsal bone in hallux valgus: a preliminary study utilizing a weight bearing extremity CT. Foot Ankle Surg. 2013;19(3):155–61. 23830162 10.1016/j.fas.2013.01.003

[23] Koivisto J, Kiljunen T, Wolff J, Kortesniemi M. Assessment of effective radiation dose of an extremity CBCT, MSCT and conventional X ray for knee area using MOSFET dosemeters. Radiat Prot Dosimetry. 2013;157(4):515–24. 23825221 10.1093/rpd/nct162

[24] Li Z, Yu L, Trzasko JD, Lake DS, Blezek DJ, Fletcher JG, et al. Adaptive nonlocal means filtering based on local noise level for CT denoising. Med Phys. 2014;41(1):011908. 24387516 10.1118/1.4851635

[25] Wolterink JM, Leiner T, Viergever MA, Isgum I. Generative adversarial networks for noise reduction in low-dose CT. IEEE Trans Med Imaging. 2017;36(12):2536–45. 28574346 10.1109/TMI.2017.2708987

[26] Ichikawa K, Kawashima H, Shimada M, Adachi T, Takata T. A three-dimensional cross-directional bilateral filter for edge-preserving noise reduction of low-dose computed tomography images. Comput Biol Med. 2019;111:103353. 31306807 10.1016/j.compbiomed.2019.103353

